# Early prediction of atherosclerosis diagnosis with medical ambient intelligence

**DOI:** 10.3389/fphys.2023.1225636

**Published:** 2023-07-20

**Authors:** Wen Yang, Qilin Nie, Yujie Sun, Danrong Zou, Jinmo Tang, Min Wang

**Affiliations:** Xiamen Hospital of Traditional Chinese Medicine, Xiamen, China

**Keywords:** atherosclerosis diagnosis, early prediction, clinical data, medical ambient intelligence, incomplete examination item

## Abstract

Atherosclerosis is a chronic vascular disease that poses a significant threat to human health. Common diagnostic methods mainly rely on active screening, which often misses the opportunity for early detection. To overcome this problem, this paper presents a novel medical ambient intelligence system for the early detection of atherosclerosis by leveraging clinical data from medical records. The system architecture includes clinical data extraction, transformation, normalization, feature selection, medical ambient computation, and predictive generation. However, the heterogeneity of examination items from different patients can degrade prediction performance. To enhance prediction performance, the “SEcond-order Classifier (SEC)” is proposed to undertake the medical ambient computation task. The first-order component and second-order cross-feature component are then consolidated and applied to the chosen feature matrix to learn the associations between the physical examination data, respectively. The prediction is lastly produced by aggregating the representations. Extensive experimental results reveal that the proposed method’s diagnostic prediction performance is superior to other state-of-the-art methods. Specifically, the Vitamin B12 indicator exhibits the strongest correlation with the early stage of atherosclerosis, while several known relevant biomarkers also demonstrate significant correlation in experimental data. The method proposed in this paper is a standalone tool, and its source code will be released in the future.

## 1 Introduction

Atherosclerosis is lipid accumulation and inflammation of the large arteries buildup, the risk pathological state of cardiovascular diseases. Cardiovascular diseases represent the major cause of mortality worldwide (Björkegren and Lusis, 2022). Atherosclerosis is a chronic vascular disease that poses a significant risk to people’s health. Early detection and active management are required to prevent its severe effects. Subclinical atherosclerosis (SA) refers to the early stage of atherosclerosis, in the aspect of clinical settings, not only signs and symptoms are subtle, but also routine examinations can not directly carry out the detection task. It makes atherosclerosis management in medicine even more challenging. Early atherosclerosis identification helps to prevent its development and lowers the risk of atherosclerosis-related diseases.

*Active screening* techniques are used for disease screening when a visitor requires certainspecific examinations. According to the nature of clinical atherosclerosis diagnosis, these methods are grouped into image-based, blood-testing, and biomarker-based methods. The image-based methods use images to identify the detailed statuses of vessels, such as doppler ultrasound (Latha et al., 2020), cardiac computed tomography angiography (Garg et al., 2022), and magnetic resonance angiography (Weinreich et al., 2017). These methods are chosen when the patient is clinically symptomatic or has risk factors. The blood-testing methods are the collection of blood from the vein, then analyzed in the laboratory to measure various components, such as plasma lipid levels (Graham et al., 2021) and coagulation tests (Koenen and Binder, 2020). Monitoring of atherosclerosis and its related complications is the purpose of these methods. The biomarker-based methods can quantify and examine the frequently particular chemicals or substances made by cells or tissues in response to the atherosclerotic process. Inflammatory biomarkers such as C-reactive protein, interleukin-6, and tumor necrosis factor-alpha, endothelial dysfunction markers such as endothelin-1 and intercellular adhesion molecule-1 (Martinez et al., 2020), but clinical settings have not yet seen the utilization of these techniques. However, in the clinical environment, these methods are frequently ignored by almost all outpatient visitors, which limits the ability to diagnose atherosclerosis early.

Computer-aided predictive methods are an emerging field of diagnostic research for disease. They not only provide decision support for diagnosing and predicting diseases (Wang et al., 2021; Huang et al., 2022), but also reveal potential factors and patterns related to disease (Zhang et al., 2022; Wang et al., 2022), which promote the innovation and development of medical knowledge. According to the nature of these methods, they are categorized into model-driven methods, data-driven methods and decision support systems (DSS). Model-driven methods apply the principles of biophysics and fluid dynamics of atherosclerosis to construct predictive models, which require a good understanding of the disease mechanisms and processes. For example, Bahloul et al. (2022) use fractional order methods to model the global and regional vessel apparent wall compliance, a biomarker of arterial stiffness, and then combined the model with carotid-to-femoral pulse wave velocity to obtain predictions. The limited knowledge of diseases and their intricacy make it challenging for researchers to set up parameters for the models. To address this issue, many studies use data-driven methods to predict atherosclerosis in its early stage. These methods rely on large-scale medical data to construct predictive models, without needing a deep knowledge of the disease mechanisms and processes. Various single or combined classification algorithms are employed to predict and diagnose atherosclerosis, such as support vector machine (SVM) (Fan et al., 2021), artificial neural network (ANN) (Terrada et al., 2019), K-nearest neighbors (KNN) (Cherradi et al., 2021), XGBoost (XGB) (Yu et al., 2021), decision tree (DT) (Chen et al., 2022) and random forest (RF) (Ward et al., 2020). However, these methods train models on specific biomarkers, which limits the generalization ability of the models. Moreover, the imbalance of medical diagnosis data of patients may impair their ability to capture existing patterns and associations in the data. DSS are systems that use computer technology and artificial intelligence methods to assist doctors or patients in making better medical decisions (Terrada et al., 2020). To apply the system to real clinical scenarios, DSS should not only use existing data for training and validation, but also consider adapting the system to clinical data, which is necessary.

The goal is to make an accurate early prediction of atherosclerosis risk, by leveraging data- and model-driven methods. There are two challenges: 1) the lack of this data type limits the performance of traditional machine learning methods. Traditional machine learning methods rely on large, diverse datasets to train models, but the lack of atherosclerosis-specific data makes it challenging to develop effective models. 2) the public or private available clinical examination data collections are quite different among patients. Since different patients may have different examination items, machine learning models that rely on these items may need to account for this variability.

To overcome the above two issues, this paper proposes an *medical ambient intelligence system (MAIS)* for the early detection of atherosclerosis. The system architecture consists of clinical record collection, data processing, model adoption, model evaluation, and decision visualization. Moreover, the *SEcond-order Classifier (SEC)* model is proposed to enhance the performance of the diagnosis task. The first-order component and second-order cross-feature component are then consolidated and applied to the chosen feature matrix in order to learn the associations between the physical examination data, respectively. The prediction is lastly produced by aggregating the representations.

The main contributions are summarized below:

1) The MAIS is proposed to predict early atherosclerosis using clinical data in medical records. The system covers the whole process of clinical application, including data processing, model adoption and system output.
2) The SEC is proposed to learn representations from sparse examined clinical items, which is suitable for common patients.
3) Experiments on clinical datasets show that the proposed SEC model achieves the performance of six state-of-the-art methods.
4) The Vitamin B12 indicator exhibits the strongest correlation with the early stage of atherosclerosis, while several known relevant biomarkers also demonstrate a significant correlation in experimental data.

The remains of this paper are organized as follows. Section 2 graphically illustrates the proposed system and the innovated diagnosis method. Section 3 gives the experimental results and their analyses. Finally, section 4 concludes this research.

## 2 Materials and methodology

### 2.1 Materials

To build a database for early prediction of atherosclerosis, it is inefficient to collect data on each patient indiscriminately and there is a lot of redundancy. Therefore, it is crucial that we precisely identify people who have a high probability of having atherosclerosis.

Nonalcoholic fatty liver disease (NAFLD) is a common chronic liver disease. It affects approximately 25% of the world’s population and is characterized by excessive accumulation of fat in the liver (Stols-Gonçalves et al., 2019). NAFLD can cause metabolic disorders such as insulin resistance, dyslipidemia, hypertension, oxidative stress, and inflammation. These are key mechanisms in the development and progression of atherosclerosis (VanWagner, 2018). People with NAFLD have been shown to have a high probability of atherosclerosis (Cattazzo et al., 2022). They often go to the liver disease centers of various hospitals for treatment. The atherosclerosis diagnosis dataset was collected from and contributed by Xiamen Hospital of Traditional Chinese Medicine Liver Disease Center.

The inclusion criteria included: First, those who signed the informed consent form, voluntarily participated in this subject, and cooperated in the completion of relevant examinations. Second, between the ages of 18 and 65 years. Third, meet the aforementioned Western diagnostic criteria for patients with NAFLD combined with SA.

Subjects who did not fit the criteria above were eliminated. The most recent report was used if a subject has undergone more than one examination because it would be more pertinent to their present condition of health.

These records contain over 1800 patients, which range from 1 January 2018, and 30 June 2022. After data extract-transform-load, the private information of patients is filtered out, and amounting to 350 examination items are picked out. Specifically, physiological information consists of urine tests, blood tests, and biochemical tests. The system automatically generates labels, using the diagnosis of SA as the basis for early detection of atherosclerosis. Subjects are labeled into two groups: positive samples with early-stage atherosclerosis (357) and negative samples without any symptoms of the disease (1491). To prevent data imbalances due to inconsistent group sizes that can affect system performance, the system selects an equal number of cases from each group: 357.

### 2.2 MAIS system

The proposed system is a novel medical ambient intelligence system that aims to provide early prediction of atherosclerosis. The system uses clinical data from medical records, such as physical and biological examinations, to build an intelligent platform compatible with various examination indicators that can classify health and pathological patterns.

According to the sequence of system execution, the system mainly consists of data processing, model adoption and system output.

**FIGURE 1:**
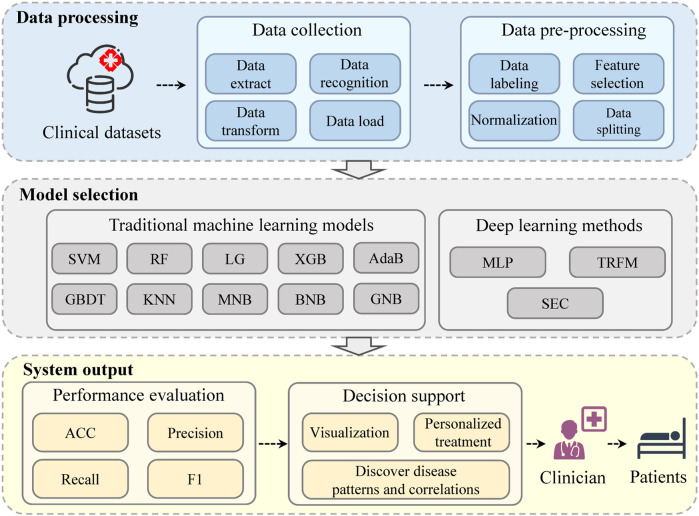
The system architecture of the medical ambient intelligence system (MAIS).

The system architecture of the proposed MAIS is graphically displayed in Figure 1.

#### 2.2.1 Data processing

In order to provide executable data to the model, the system first performs data processing. The system needs data in real clinical scenarios, which can be obtained by connecting to the database of the medical system. The data of patients’ clinical routine examination items are both structured and unstructured, including patient demographics, medical history, laboratory test results, imaging reports, and other relevant clinical information.

The system automatically performs data extract, transform, load (ETL) (Manickam and Indra, 2021) processing and data recognition in the data collection stage. It uses natural language processing (NLP) (Galassi et al., 2021) techniques to extract relevant information from unstructured data, such as personal details, medical records, risk factors, symptoms, diagnoses and treatments. The system also applies named entity recognition (NER) (Li et al., 2022) and coreference resolution (CR) (Stylianou and Vlahavas, 2021) to deal with inconsistent representations in unstructured text data. For structured data, the system uses data manipulation tools to extract the necessary fields or tables from the data. Then, it transforms the extracted data into a common representation that can be used by the subsequent modules, and loads it into a centralized data repository.

In the data preprocessing stage, the diagnosis label for each patient is automatically generated in the system, using the diagnosis of subclinical atherosclerosis as the basis for early detection of atherosclerosis. The system then performs normalization, feature selection, and splits the data into training and testing sets.

#### 2.2.2 Model adoption

As an important part of the medical ambient computation, model adoption is to select the best model for the given data and task. The system applies ten traditional machine learning models and three deep learning models for the early prediction of atherosclerosis.

The baseline methods used to evaluate the effectiveness of the SEC. Some typical machine learning methods are adopted as parts of baseline methods:

1) Support vector machine (SVM) (Dudzik et al., 2021) projects the features into high dimensionality and finds the optimal hyperplane to generate classifications.
2) Random forest (RF) (Probst and Boulesteix, 2018) builds multiple decision trees via randomly selecting features and generating classification results by tree voting.
3) Logistic regression (LG) (Shipe et al., 2019) is a type of statistical model that estimates the probability of an event occurring.
4) K-nearest neighbors (KNN) (Cherradi et al., 2021) is a non-parametric method, which uses the proximity of the k closest training examples in a data set to classify.
5) Gradient boost decision tree (GBDT) (Si et al., 2017) is an iterative decision tree algorithm that generates classifications via multiple decision trees.
6) XGBoost (XGB) (Munkhdalai et al., 2020) optimizes the loss function by fitting the annotations and the residuals.
7) Gaussian naive bayes (GNB), multinomial naive bayes (MNB) and Bernoulli naive bayes (BNB) (Dou et al., 2022) are all Bayes-based methods, and the differences between them are the likelihood estimation of features.
8) Adaboost (AdaB) (Shihua et al., 2020) adds a new weak classifier in each round until a predetermined small enough error rate is reached.

Some classical deep learning methods are adopted as parts of baseline methods as well:

9) Multilayer perceptron (MLP) (Okanoue et al., 2021) maps the features through the multi-layer hidden neural to minimize the loss function.
10) Transformer (TRMF) (Vaswani et al., 2017) is a variation of the encoder-decoder structure, which replaces the recurrent layer with the attention mechanism.

#### 2.2.3 System output

The system output has two main components: performance evaluation and decision support. The performance evaluation measures the accuracy and reliability of the system model on various metrics. The decision support offers useful information and guidance to clinicians, such as data and results in visualization, disease pattern and correlation discovery, and personalized treatment suggestions.

### 2.3 SEcond-order classifier

#### 2.3.1 Problem definition

The SA diagnosis problem can be considered a binary classification problem. Let symbol $X\in R^{N\times M}$ represent the test items matrix, where *N* represents the number of samples, and symbol *M* is the number of test items. The binary classification problem can be formulated as follows:

$$Y\leftarrow F\left(X\right)$$ (1)

where $Y\in R^{N}$ is the classification target, *F* (⋅) is a mapping.

#### 2.3.2 Overview

**FIGURE 2:**
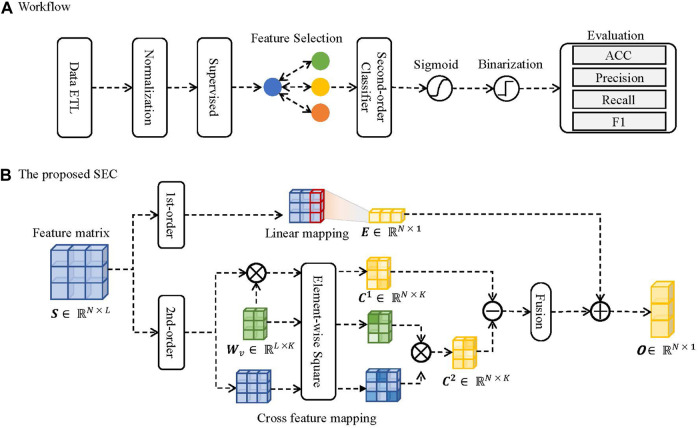
The schematic illustration of the proposed SEC. **(A)** The workflow. **(B)** The proposed SEC.

Firstly, the inputted data are compressed into [0, 1] by the normalization layer in the proposed SEC. And it was then passed through a feature selection layer for dimension reduction. After that, the selected feature matrix is fed to the proposed SEC to connect with classification targets. Finally, a sigmoid function and a binarization function are employed on the outputs to generate classifications. The graphical illustration of the proposed SEC is plotted in Figure 2.

#### 2.3.3 Data preprocessing

The *MinMax-normalization* (Singh and Singh, 2020) is employed to compress the inputted features into [0, 1]. The mathematical process of normalization is formulated as follows:

$$Z=\frac{X-\min\left(X\right)}{\max\left(X\right)-\min\left(X\right)}$$ (2)

where ***Z*** is the normalized feature matrix, ***X*** is the inputted feature matrix, min (⋅) is the minimum values, and max (⋅) is the maximum values.

The *feature selection* (Dhal and Azad, 2021) is utilized for dimension reduction. The “ANOVA F-value” is chosen as the ranking criteria for feature selection. The mathematical process is formulated as follows:

$$f\propto \frac{{\left(\sum_{i}^{N}Z_{i}^{0}-\sum_{i}^{N}Z_{i}^{1}\right)}^{2}}{\left(\sum_{i}^{N}\sqrt{Z_{i}^{0}}+\sum_{i}^{N}\sqrt{Z_{i}^{1}}\right)-2{\left(\sum_{i}^{N}Z_{i}^{0}\right)}^{2}-2{\left(\sum_{i}^{N}Z_{i}^{1}\right)}^{2}}$$ (3)

where $f\in R^{M}$ is the F-value matrix of inputted features, ***Z*** ^0^ is the samples without SA, ***Z*** ^1^ is the samples diagnosed with SA. And then, the F-value matrix is consolidated with the survival function to rank and select the best *L* features. Let symbol $S\in R^{N\times L}$ represent the selected feature matrix.

#### 2.3.4 Workflow

The proposed SEC is constituted of two parts. The first component is first-order linear regression, designed to capture associations between prediction and input features. Another part is the cross-association component, which captures the associations between features. The outputs come from the combinations of the two components.

Given an inputted feature matrix $S\in R^{N\times L}$ , the mathematical process of the first-order linear regression is formulated as follows:

$$E=\sum \left(S⊙W_{e}\right)+B_{e}$$ (4)

where $E\in R^{N\times 1}$ is the outputs of linear layer, ***W***_*e*_ is the weight matrix, *B*_*e*_ is a bias term, ⊙ is Hadamard product.

The given inputted feature matrix ***S*** collaborates with a learnable hidden feature matrix ***W***_*v*_ to calculate the second-order cross features to learn the feature associations. The mathematical processes are formulated as follows:

$$C^{1}=\left(S\cdot W_{v}\right)⊙\left(S\cdot W_{v}\right)$$ (5)

$$C^{2}=\left(S⊙S\right)\cdot \left(W_{v}⊙W_{v}\right)$$ (6)

where $C^{1}\in R^{N\times K}$ is the square of cross feature matrix, $C^{2}\in R^{N\times K}$ is the square-cross feature matrix, $W_{v}\in R^{L\times K}$ , *K* is the number of hidden features.

***C*** ^1^ and ***C*** ^2^ are subtracted to generate hidden feature representations, and the different hidden features are summed to generate second-order outputs. The mathematical process is formulated as follows:

$$R=\frac{1}{2}\overset{K}{\underset{i}{\sum }}\left(C_{n,i}^{1}-C_{n,i}^{2}\right)$$ (7)

where $R\in R^{N\times 1}$ is the hidden feature representations.

The ***T*** and ***R*** are integrated to generate model outputs. The mathematical process is formulated as follows:

O=E+R (8)

where $O\in R^{N\times 1}$ is the outputs of the model.

Finally, the output matrix ***O*** is passed through a sigmoid layer and a binarization layer to generate classifications. The mathematical process can be formulated as follows:

$$P_{i}=\frac{1}{1+\exp\left(-O_{i}\right)}$$ (9)

$$Y_{i}=\left\{\begin{array}{cc} & 0,\;P_{i}<0.5\\ & 1,\;P_{i}\geq 0.5 \end{array}\right.$$ (10)

where $P\in R^{N\times 1}$ is the probability matrix, $Y\in R^{N\times 1}$ is the binary output matrix.

### 2.4 Performance criteria

The *accuracy*, *precision*, *recall*, and *F*1 are chosen as the metrics for assessing the effectiveness of the proposed SEC and baseline methods. The mathematical formulation of these methods and corresponding formulations are listed in Table 1 and Table 2:

**TABLE 1:** Confusion matrix for binary classification.

Real	False	True
Predicted		
False	FP	TN
True	FN	TP

**TABLE 2:** Evaluation metrics of classification performance for the proposed method and benchmarks.

Metrics	Formula
Accuracy (ACC)	$\frac{TP+TN}{TP+TN+FP+FN}$
Precision	$\frac{TP}{TP+FP}$
Recall	$\frac{TP}{TP+TN}$
F1-Means (F1)	$\frac{2\cdot Precision\;\cdot \;Recall}{Precision\;+\;Recall}$

### 2.5 Model configurations

Data processed in the same way is fed to different models to ensure a fair and unbiased comparison between them. The data are all compressed into [0, 1] using the MinMax normalization technique. The ANOVA F-value is used as the ranking criterion for feature selection, and the best L features are selected according to their F-value. The 80% data is utilized for training the models, and the remains are used to test the trained models. The five-fold cross-validation strategy is employed to validate the performance of baseline methods and the proposed SEC. The “Adam” is chosen as the optimizer for all neural models. The batch size is set to 32, and the learning rate is set to 0.01. The “binary cross entropy loss with logits” (BCELosswithLogits) is selected as the loss function. For the ensemble learning-based methods, the number of base estimators is set to 200.

## 3 Results and discussion

### 3.1 Performance comparison

**FIGURE 3:**
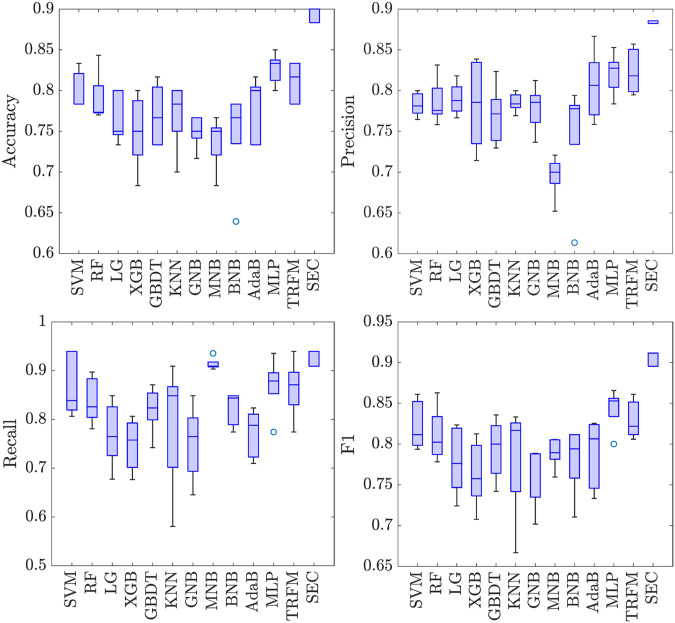
Five-fold cross-validation of eleven methods in terms of four metrics.

Extensive experiments are carried out to examine the effectiveness of the suggested SEC. For all models, the constant parameters are set to be the same, and the other parameter is well-tuned. The experimental results are plotted in Figure 3.

Here is a summary of some findings from the experiments:

1) The proposed SEC outperforms the baseline methods.
2) Among baseline approaches, the Bayes-based methods produce the worst results.
3) SVM outperforms other machine learning techniques in terms of performance.
4) When compared to machine learning methods, deep learning techniques offer tremendous advances.

The Bayes-based methods achieve worse performance. A possible reason is that these methods decide a specific joint probability distribution. However, due to some effects, such as missing value or sparsity, the distribution of the inputted features does not follow the Gaussian or Bernoulli distribution.

The SVM obtains the highest mean average values of *Accuray*, *Recall*, and *F*1. Moreover, it obtains a smaller interquartile range compared with other machine learning methods. A possible reason is that this method gets the final classification via a few support vectors. It is beneficial for the model to learn key associations from inputted features.

The deep learning methods outperform machine learning methods. The multilayer perceptron (MLP) and transformer (TRMF) have higher average values of *ACC*, *P*, *R*, and *F*1. Moreover, they have a smaller interquartile range compared with machine learning methods. It demonstrates that these methods have robust performance. A significant reason is that machine learning methods usually get classification via a few key features or representations. However, these may ignore some beneficial effects of some features.

The proposed SEC has significant improvements compared with the baseline methods. Compared with the optimal performance of baseline methods, the *ACC*, *Precision*, *Recall*, and *F*1 value is increased by 7.12%, 7.12%, 0.81%, 8.74% at least, respectively. Moreover, the SEC has the smallest interquartile range among all comparable methods, which shows the robustness of the proposed method.

### 3.2 Parameter sensitiveness

**FIGURE 4:**
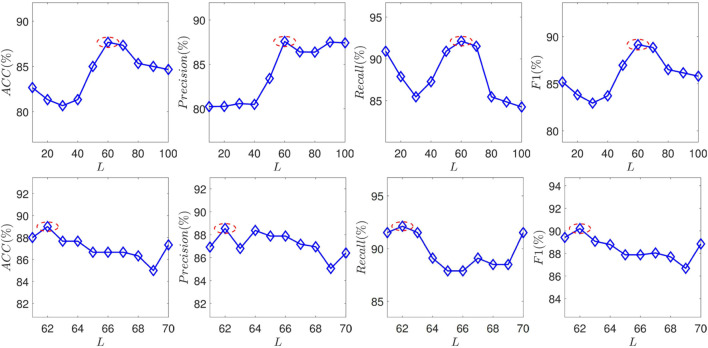
The performance of the proposed SEC with varying the feature number *N* in terms of *ACC*, *Precision*, *Recall*, and *F*1.

To investigate how these features affect the classification performance, the proposed SEC are measured by *ACC*, *Precision*, *Recall*, and *F*1 by varying the number of selected features. There is an extensive range of feature numbers. To avoid searching for all situations, the coarse-tune and fine-tune are combined to search for the optimal *L*. Technically, the parameter first adds 10 features per varying until the model shows a noticeable decrease. And then, the parameter adds one feature per varying until the optimal performance is found. The experimental results are plotted in Figure 4. And the selected features are listed in Table 3.

**TABLE 3:** The selected physical examination items and their average values.

Category	Name	Unit	Abbr	Mean
Viral hepatitis	Hepatitis B virus e antibody	PEIU/mL	anti-HBe	0.0303
	Hepatitis B virus core antibody	PEIU/mL	anti-HBc	0.3748
	Hepatitis B virus surface antibody	mIU/mL	anti-HBs	5.4965
	Hepatitis B virus DeoxyriboNucleicAcid	IU/mL	HBV-DNA	2880.0767
	Hepatitis C virus antibody	COI	anti-HCV	0.0013
	Hepatitis A virus antibody Immunoglobulin M	S/CO	HAV-IgM	0.0157
Glucose tolerance test	Half of hour blood glucose	mmol/L	0.5 h BG	0.1670
	One hour blood glucose	mmol/L	1 h BG	0.1899
	Two hour blood glucose	mmol/L	2 h BG	0.3355
Inflammation	Procalcitonin	ng/mL	PCT	0.0392
Thromboela-stogram	Fibrin polymerization time	s	Fbg-time	0.1093
	Coagulation factor response time	s	Cfr-time	0.3940
	Residual blood clots after drug use	mm	Rbc-adu	1.6047
	Maximum platelet aggregation rate	%	PAGM	0.5707
	Mean platelet aggregation rate	%	PARM	0.5360
	Mean platelet aggregation time	s	PATM	4.2400
	Platelet	mm	MA	4.1583
	Clot ablation ratio	%	CAR	0.0383
	Predicted clot ablation rate	%	PCAR	0.0430
	Platelet effective inhibition rate	%	PRU	0.4293
	Instantaneous amplitude on trace	mm	A	4.1453
	Conductivity	S/m	Gt	11.1207
	Fubction of Fibrin Monomer	deg		4.4740
Blood routine examination	Neutrophil granulocyte	%	NEUT%	55.5910
	Erythrocyte/Red Blood Cell	km	RBC	6.3503
	Leukocyte, white blood cell	/L	WBC	10.1733
	Hemoglobin/Haemoglobin	g/L	HGB	124.3500
	*Erythrocyte Count (×10^12^)	/L	RBC	4.1514
	Erythrocyte Count (×10^10^)	/L	RBC	3.8233
	Mean corpuscular volume	FL	MCV	0.9643
	Mean platelet volume	fl	MPV	9.3843
	Initial mean volume of platelets	fl	IMPV	0.0819
	Mean corpuscular volume	FL	MCV	0.9643
	Packed cell volume	%	PCV	36.5337
	Mean red blood cell hemoglobin concentration	g/L	MCHC	307.1667
Routine blood biochemical	Triglyceride	mmol/L	TG	2.2369
	Albumin	g/L	Alb	37.1433
	Total Protein	g/L	TP	64.3200
	Uric acid	mg	UA	328.5420
	*γ*-glutamyl transpeptidase	U/L	*γ*-GT	59.8733
	Liver function leukocyte ratio	None	A/G	1.2977
	haptoglobin	haptoglobin	HP	0.0173
	a1_acid glycoprotein	mg/L	a1-AG	0.0077
	Carbohydrate antigen	u/mL	CA	6602.2316
	Thyroglobulin	ug/L	Tg	0.1275
	ImmunoglobulinM	g/L	IgM	0.0338
	Vitamin B	mg	VB	82.2867
	Sodium	mmol/L	Na	0.8883
	Potassium Kalium	mmol/L	K	0.2110
Blood gas analysis	Carbon dioxide	g/L	CO_2_	21.7383
	2-Hydroxypropanoic acid	mmol/L	HL	0.1675
Adrenocortical	Angiotensin II	ng/dL	Ang II	0.5947
	Aldosterone	ng/dL	ALD,ALS	1.2386
HIV	HIV antigens/antibodies	COI	HIV-Ag/HIV-Ab	0.0410
	HIV antigens/antibodies	COI	HIV-Ag/HIV-Ab	0.0002
Antiphospholipid syndrome	Anti-beta 2 glycoprotein 1 antibody	RU/mL	anti-B2-GPI ab	0.0074
	Anti cardiolipin antibody Immunoglobulin M	MPLU/mL	ACA-IgM	0.0361
	Anti-dsDNA antibody	IU/mL	Anti-dsDNA ab	0.1024
Heart failure	N-terminal pro-brain natriuretic peptide	PGml	NT-pro BNP	26.9633
Myocardial damage	Myoglobin	g/L	Mb	31.5280
Urine test	Erythrocyte	HPF	RBC	1.1430
Urine culture	Urine culture	HPF	UC-CJ	53.2273
	Urine culture	uL	UC	295.7010

Some observations from the experimental results are summarized as follows:

1) Vitamin B12 is the most correlated with SA.
2) Several conventional related biomarkers show strong correlations in the experiments.
3) Some pathogen-related metrics show associations with SA.
4) Some NAFLD-related metrics exhibit correlations with SA.

Vitamin B12 correlates significantly with the SA. Besides, several studies have reported that vitamin B can alleviate the burden of the SA (Hodis et al., 2009) It implies that vitamin B may be a criterion for the SA diagnosis within NAFLD patients.

Some established metrics have been identified, including thrombus-related biomarkers and markers of platelet reactivity. However, as shown in Figure 4, the model cannot achieve optimal performance solely using these metrics. It demonstrates that these metrics can not wholly represent the effects of SA. Biomarkers for viral hepatitis and measures for HIV are two examples of pathogen-related metrics that have a good correlation with model performance. There are two potential reasons: First, the patients infected with these infectious diseases may have a higher risk of suffering from SA. For example, the incidence of coronary plaque in people infected with HIV is 2–3 times higher than in healthy people (Boldeanu et al., 2021). On the other hand, a possible reason is the collected dataset. SA commonly has no significant symptoms. Some patients may be diagnosed with atherosclerosis incidentally due to infectious diseases.

Several glucose-tolerance-test metrics have been selected, such as half-hour, 1-h, and 2-h blood glucose. It demonstrates that the anomaly of these factors would bring a higher risk for SA. These factors are also risky for the NAFLD. The balance of lipid-glucose metabolism may be destroyed by NAFLD, which increases the risk of SA. This experiment might support this assertion further.

### 3.3 N-order analyses

They are solely employed for the classification task to study how each order affects performance. The experimental results are shown in Table 4.

**TABLE 4:** The experimental results of different order in terms of four metrics.

Model	ACC	P	R	F1
First-Order linear regression	0.8067	0.8022	0.8606	0.8303
Second-Order cross feature	0.8167	0.8362	0.8303	0.8327
Full SEC	0.8900	0.8837	0.9212	0.9020

As shown in Table 4, solely using one of the components cannot achieve the optimal prediction performance. These two components obtain similar prediction accuracy. The first-order linear regression is mainly employed to learn the associations between inputted features and the prediction targets. And the second-order cross-feature component is mainly utilized to learn the associations between inputted features. The combination of the two-component has a significant increase in prediction accuracy. Compared with the optimal performance of component, the *ACC*, *Precision*, *Recall* and *F*1 values are increased by 8.98%, 5.68%, 10.95% and 8.32%, respectively. It shows that the full SEC can learn the correlations between inputted features and prediction targets well.

## 4 Conclusion

This paper proposed the MAIS for the early prediction of atherosclerosis disease. This system supports the early screening and discovery of outpatients in the real environment. Moreover, the SEC model is proposed to enhance the prediction performance of atherosclerosis diagnosis under the heterogeneity of clinical data. The proposed combination of first-order components and second-order cross-feature components learns effective associations and representations from sparsely examined clinical items.

Extensive experiments are conducted on real atherosclerosis check item datasets to explore the effectiveness of the proposed method. Compared with baseline, *ACC*, *Precision*, *Recall*, and *F*1 values increased by at least 7.12%, 7.12%, 0.81%, 8.74%, respectively. Furthermore, each order in SEC is divided to perform the classification tasks independently. The results demonstrate that the full SEC is capable of learning the relationships between input features and prediction targets, and when compared to the component’s optimal performance, the *ACC*, *Precision*, *Recall*, and *F*1 values all show increases of 8.98%, 5.68%, 10.95%, and 8.32%, respectively. In addition, the experimental analysis showed that the Vitamin B12 indicator exhibits the strongest correlation with the early stage of atherosclerosis, while several known relevant biomarkers also demonstrate a significant correlation in experimental data.

There are several directions for future work. First, the validation data utilized in this study are somewhat constrained. Our intended strategy involves gathering a more diverse array of data spanning various geographical regions and demographic profiles. This could significantly enhance the universal applicability of the system and improve its generalizability to disparate demographics and locales. Second, we aspire to elucidate the relevance of exogenous features by incorporating efficacious feature selection and fusion methodologies. This approach might strengthen interpretability during the process of feature learning. Third, the purview of the system should extend beyond atherosclerosis, encompassing additional diseases. Concurrently, there is a need to investigate how medical environmental intelligence can be systematically exploited to predict and diagnose various diseases.

## Data Availability

The data analyzed in this study is subject to the following licenses/restrictions: The data used to support the findings of this study are available from the corresponding authors upon request. Requests to access these datasets should be directed to ywen99012@gmail.com.

## References

[B1] Bahloul M. Belkhatir Z. Aboelkassem Y. Laleg-Kirati T.-M. (2022). Physics-based modeling and data-driven algorithm for prediction and diagnosis of atherosclerosis. Biophysical J. 121, 419a–420a. 10.1016/j.bpj.2021.11.653

[B2] Björkegren J. L. Lusis A. J. (2022). Atherosclerosis: Recent developments. Cell 185, 1630–1645. 10.1016/j.cell.2022.04.004 35504280 PMC9119695

[B3] Boldeanu I. Sadouni M. Mansour S. Baril J.-G. Trottier B. Soulez G. (2021). Prevalence and characterization of subclinical coronary atherosclerotic plaque with ct among individuals with hiv: Results from the canadian hiv and aging cohort study. Radiology 299, 571–580. 10.1148/radiol.2021203297 33876969

[B4] Cattazzo F. Lombardi R. Mantovani A. Bevilacqua M. Zoncapè M. Prat L. I. (2022). Subclinical and clinical atherosclerosis in non-alcoholic fatty liver disease is associated with the presence of hypertension. Nutr. Metab. Cardiovasc. Dis. 32, 2839–2847. 10.1016/j.numecd.2022.08.005 36404479

[B5] Chen Z. Yang M. Wen Y. Jiang S. Liu W. Huang H. (2022). Prediction of atherosclerosis using machine learning based on operations research. Math. Biosci. Eng. 19, 4892–4910. 10.3934/mbe.2022229 35430846

[B6] Cherradi B. Terrada O. Ouhmida A. Hamida S. Raihani A. Bouattane O. (2021). “Computer-aided diagnosis system for early prediction of atherosclerosis using machine learning and k-fold cross-validation,” in 2021 International Congress of Advanced Technology and Engineering (ICOTEN) (IEEE), 1–9.

[B7] Dhal P. Azad C. (2021). A comprehensive survey on feature selection in the various fields of machine learning. Appl. Intell. 52, 4543–4581. 10.1007/s10489-021-02550-9

[B8] Dou H. Tan J. Wei H. Wang F. Yang J. Ma X.-G. (2022). Transfer inhibitory potency prediction to binary classification: A model only needs a small training set. Comput. Methods Programs Biomed. 215, 106633. 10.1016/j.cmpb.2022.106633 35091229

[B9] Dudzik W. Nalepa J. Kawulok M. (2021). Evolving data-adaptive support vector machines for binary classification. Knowledge-Based Syst. 227, 107221. 10.1016/j.knosys.2021.107221

[B10] Fan J. Chen M. Luo J. Yang S. Shi J. Yao Q. (2021). The prediction of asymptomatic carotid atherosclerosis with electronic health records: A comparative study of six machine learning models. BMC Med. Inf. Decis. Mak. 21, 115. 10.1186/s12911-021-01480-3 PMC8020544 33820531

[B11] Galassi A. Lippi M. Torroni P. (2021). Attention in natural language processing. IEEE Trans. Neural Netw. Learn. Syst. 32, 4291–4308. 10.1109/TNNLS.2020.3019893 32915750

[B12] Garg K. Patel T. R. Kanwal A. Villines T. C. Aggarwal N. R. Nasir K. (2022). The evolving role of coronary computed tomography in understanding sex differences in coronary atherosclerosis. J. Cardiovasc. Comput. Tomogr. 16, 138–149. 10.1016/j.jcct.2021.09.004 34654676 PMC9358989

[B13] Graham S. E. Clarke S. L. Wu K.-H. H. Kanoni S. Zajac G. J. Ramdas S. (2021). The power of genetic diversity in genome-wide association studies of lipids. Nature 600, 675–679. 10.1038/s41586-021-04064-3 34887591 PMC8730582

[B14] Hodis H. N. Mack W. J. Dustin L. Mahrer P. R. Azen S. P. Detrano R. (2009). High-dose B vitamin supplementation and progression of subclinical atherosclerosis: A randomized controlled trial. Stroke 40, 730–736. 10.1161/STROKEAHA.108.526798 19118243 PMC2701290

[B15] Huang Y. Zhang P. Wang Z. Lu Z. Wang Z. (2022). HFMD cases prediction using transfer one-step-ahead learning. Neural Process. Lett. Early View. 10.1007/s11063-022-10795-9

[B16] Koenen R. R. Binder C. J. (2020). Platelets and coagulation factors: Established and novel roles in atherosclerosis and atherothrombosis. Atherosclerosis 307, 78–79. 10.1016/j.atherosclerosis.2020.07.008 32718764

[B17] Latha S. Samiappan D. Kumar R. (2020). Carotid artery ultrasound image analysis: A review of the literature. Proc. institution Mech. Eng. Part H J. Eng. Med. 234, 417–443. 10.1177/0954411919900720 31960771

[B18] Li J. Sun A. Han J. Li C. (2022). A survey on deep learning for named entity recognition. IEEE Trans. Knowl. Data Eng. 34, 50–70. 10.1109/tkde.2020.2981314

[B19] Manickam V. Indra M. R. (2021). Dynamic multi-variant relational scheme-based intelligent etl framework for healthcare management. Soft Comput. 27, 605–614. 10.1007/s00500-022-06938-8 PMC8935255 35340776

[B20] Martinez E. Martorell J. Riambau V. (2020). Review of serum biomarkers in carotid atherosclerosis. J. Vasc. Surg. 71, 329–341. 10.1016/j.jvs.2019.04.488 31327598

[B21] Munkhdalai L. Munkhdalai T. Ryu K. H. (2020). Gev-nn: A deep neural network architecture for class imbalance problem in binary classification. Knowledge-Based Syst. 194, 105534. 10.1016/j.knosys.2020.105534

[B22] Okanoue T. Shima T. Mitsumoto Y. Umemura A. Yamaguchi K. Itoh Y. (2021). Artificial intelligence/neural network system for the screening of nonalcoholic fatty liver disease and nonalcoholic steatohepatitis. Hepatology Res. 51, 554–569. 10.1111/hepr.13628 33594747

[B23] Probst P. Boulesteix A.-L. (2018). To tune or not to tune the number of trees in random forest. J. Mach. Learn. Res. 18, 1–18.

[B24] Shihua L. Zian D. Tianxin C. Hongyi C. Ling J. (2020). A weighted svm ensemble predictor based on adaboost for blast furnace ironmaking process. Appl. Intell. 50, 1997–2008. 10.1007/s10489-020-01662-y

[B25] Shipe M. E. Deppen S. A. Farjah F. Grogan E. L. (2019). Developing prediction models for clinical use using logistic regression: An overview. J. Thorac. Dis. 11, S574–S584. 10.21037/jtd.2019.01.25 31032076 PMC6465431

[B26] Si S. Zhang H. Keerthi S. S. Mahajan D. Dhillon I. S. Hsieh C.-J. (2017). “Gradient boosted decision trees for high dimensional sparse output,” in Proceedings of the 34th International Conference on Machine Learning (Sydney, Australia: PMLR), 3182–3190.70

[B27] Singh D. Singh B. (2020). Investigating the impact of data normalization on classification performance. Appl. Soft Comput. 97, 105524. 10.1016/j.asoc.2019.105524

[B28] Stols-Gonçalves D. Hovingh G. K. Nieuwdorp M. Holleboom A. G. (2019). Nafld and atherosclerosis: Two sides of the same dysmetabolic coin? Trends Endocrinol. Metabolism 30, 891–902. 10.1016/j.tem.2019.08.008 31630897

[B29] Stylianou N. Vlahavas I. (2021). A neural entity coreference resolution review. Expert Syst. Appl. 168, 114466. 10.1016/j.eswa.2020.114466

[B30] Terrada O. Cherradi B. Raihani A. Bouattane O. (2019). “Classification and prediction of atherosclerosis diseases using machine learning algorithms,” in 2019 5th International Conference on Optimization and Applications (Kenitra, Morocco: IEEE), 1–5.

[B31] Terrada O. Cherradi B. Raihani A. Bouattane O. (2020). A novel medical diagnosis support system for predicting patients with atherosclerosis diseases. Inf. Med. Unlocked 21, 100483. 10.1016/j.imu.2020.100483

[B32] VanWagner L. B. (2018). New insights into NAFLD and subclinical coronary atherosclerosis. J. Hepatol. 68, 890–892. 10.1016/j.jhep.2018.01.023 29410378 PMC5903205

[B33] Vaswani A. Shazeer N. Parmar N. Uszkoreit J. Jones L. Gomez A. N. (2017). “Attention is all you need,” in Proceedings of the 31th International Conference on Neural Information Processing Systems (Long Beach, CA, USA: Curran Associates, Inc.), 5998–6008.

[B34] Wang Z. Huang Y. He B. (2021). Dual-grained representation for hand, foot, and mouth disease prediction within public health cyber-physical systems. Softw. Pract. Exp. 51, 2290–2305. 10.1002/spe.2940

[B35] Wang Z. Wang Z. Lu L. Huang Y. Fu Y. (2022). A multi-view multi-omics model for cancer drug response prediction. Appl. Intell. 52, 14639–14650. 10.1007/s10489-022-03294-w

[B36] Ward A. Sarraju A. Chung S. Li J. Harrington R. Heidenreich P. (2020). Machine learning and atherosclerotic cardiovascular disease risk prediction in a multi-ethnic population. NPJ Digit. Med. 3, 125. 10.1038/s41746-020-00331-1 33043149 PMC7511400

[B37] Weinreich M. Litwok Y. Mui L. W. Lau J. F. (2017). Advanced vascular imaging. Vasc. Med. 22, 73–76. 10.1177/1358863X16681666 28215105

[B38] Yu J. Zhou Y. Yang Q. Liu X. Huang L. Yu P. (2021). Machine learning models for screening carotid atherosclerosis in asymptomatic adults. Sci. Rep. 11, 22236. 10.1038/s41598-021-01456-3 34782634 PMC8593081

[B39] Zhang P. Wang Z. Huang Y. Wang M. (2022). Dual-grained directional representation for infectious disease case prediction. Knowledge-Based Syst. 256, 109806. 10.1016/j.knosys.2022.109806

