# Supplementary material for: Early prediction of atherosclerosis diagnosis with medical ambient intelligence
Source: Front Physiol. 2023 Jul 20;14:1225636. doi: 10.3389/fphys.2023.1225636 (PMC10398961; doi:10.3389/fphys.2023.1225636)
Supplement: Supplementary file 1 [file Table5.pdf]

Supplementary Material

1 SUPPLEMENTARY TABLES

Table S1. The main notations used.

Notation	Meaning
N	Samples number
M	Test items number
L	Selected test items number
K	Hidden feature number
$\boldsymbol{X}$	Input matrix, where $\boldsymbol{X} \in \mathbb{R}^{N\times M}$
$\boldsymbol{Z}$	Normalized input matrix, $\boldsymbol{Z} \in \mathbb{R}^{N\times M}$
$\boldsymbol{S}$	Selected input matrix $\boldsymbol{S} \in \mathbb{R}^{N\times L}$
$\boldsymbol{W}_e$	Linear weight matrix $\boldsymbol{W}_e \in \mathbb{R}^{L\times 1}$
$\boldsymbol{W}_v$	Hidden feature weight matrix $\boldsymbol{W}_v \in \mathbb{R}^{L\times K}$
$\boldsymbol{O}$	Outputs matrix $\boldsymbol{O} \in \mathbb{R}^{N\times 1}$
$\boldsymbol{P}$	Probability matrix, $\boldsymbol{P} \in \mathbb{R}^{N\times 1}$
$\boldsymbol{Y}$	Binary target matrix, $\boldsymbol{Y} \in \mathbb{R}^{N\times 1}$

Table S2. Corresponding meanings and categories of acronyms.

Abbreviation	Meaning	Category
NAFLD	Nonalcoholic fatty liver disease	Concept
SA	Subclinical atherosclerosis	Concept
DSS	Decision support systems	Concept
ETL	Extract, transform, load	Concept
NLP	Natural language processing	Concept
NER	Named entity recognition	Concept
CR	Coreference resolution	Concept
SVM	Support vector machine	Model
RF	Directional representation extraction	Model
LG	Logistic regression	Model
KNN	K-nearest neighbors	Model
GBDT	Gradient boosting decision tree	Model
XGB	Directional representation unit	Model
GNB	Gaussian naive bayes	Model
MNB	Multinomial naive bayes	Model
BNB	Bernoulli naive bayes	Model
ADA	Adaboost	Model
MLP	Multi-layer perceptron	Model
TRMF	Transformer	Model
